# Intrahepatic cholangiocarcinoma in the setting of HBV-related cirrhosis: Differentiation with hepatocellular carcinoma by using Intravoxel incoherent motion diffusion-weighted MR imaging

**DOI:** 10.18632/oncotarget.23807

**Published:** 2017-12-26

**Authors:** Yi Wei, Feifei Gao, Dandan Zheng, Zixing Huang, Min Wang, Fubi Hu, Chenyang Chen, Ting Duan, Jie Chen, Likun Cao, Bin Song

**Affiliations:** ^1^ Department of Radiology, West China Hospital, Sichuan University, Chengdu, China; ^2^ Department of Radiology, Henan Provincial People's Hospital, Zhengzhou, China; ^3^ GE Healthcare China, Beijing, China

**Keywords:** intravoxel incoherent motion, diffusion weighted imaging, hepatocellular carcinoma, intrahepatic cholangiocarcinoma

## Abstract

Accurate preoperative differentiation of intrahepatic cholangiocarcinoma (ICC) and hepatocellular carcinoma (HCC) in the setting of cirrhotic liver is of great clinical significance because the treatment and prognosis of these entities differ markedly. Through a retrospectively research, we sought to determine the diagnostic performances of intravoxel incoherent motion (IVIM) and diffusion weighted imaging (DWI) parameters in the differentiating of ICC and HCC. According to the results, we found that apparent diffusion coefficient (ADC) derived from mono-exponential model and true ADC (ADC^slow^) derived from bi-exponential model can be used to distinguish the ICC and HCC, and ADC^slow^entailed the higher diagnostic performance than ADC. However, pseudo-ADC (ADC^fast^) and perfusion fraction (f) can not be used to differentiate ICC and HCC. These results suggested that IVIM and DWI parameters can be useful in differentiating ICC and HCC and might be helpful in selecting the treatment plan and predicting prognosis.

## INTRODUCTION

Intrahepatic cholangiocarcinoma (ICC), an aggressive epithelial malignancy of the bile ducts [1], is the second most common primary liver cancer worldwide, with a rising incidence [2]. The overall prognosis of ICC remains poor with the five-year survival rate less than 30% [3]. Due to the common risk factor of Hepatitis-B virus (HBV) infection with hepatocellular carcinoma (HCC), ICC can also be presented with cirrhosis [4, 5]. Differentiation of ICC and HCC in the setting of liver cirrhosis is of great importance, because the treatment and prognosis of these entities can be quite different [6, 7]. Ultrasonography (US) is helpful to investigate the cause of the bile duct obstruction and to locate the lesions [8, 9], computed tomography (CT) is usually used to evaluate the full extension of tumor and determine surgical resectability [10, 11], routine unenhanced T1- and T2-weighted imaging enable the ability to evaluate the surrounding tissues, magnetic resonance cholangiopancreatography (MRCP) is useful for assessing the biliary system [12–14]. However, accurate preoperative diagnosis of ICC in patients with cirrhosis has been quite difficult by using these usual imaging because of similar imaging features compared with HCC especially in the cirrhotic liver [15].

Diffusion weighted imaging (DWI) is a noninvasive approach to probe molecular diffusion of water without contrast administration, and the diffusion of water can be quantitatively described by apparent diffusion coefficient (ADC) [16, 17]. However, the ADC value derived from DWI is calculated by using the mono-exponential model, which ignored the effect of perfusion fraction in tissue and could be influenced by the microcirculation of blood in capillaries [18, 19]. Intravoxel incoherent motion (IVIM) diffusion-weighted MR imaging could determine the true molecular diffusion and perfusion from the blood microcirculation in the capillary networks by using multi *b* values, and thus has the potential to better characterize tissue than DWI [20, 21]. To our knowledge, there is no report that have compared IVIM-DWI derived from the bio-exponential model and conventional DWI derived from mono-exponential model on their capabilities to differentiate ICC and HCC in cirrhotic livers.

The purpose of this study was to determine the feasibility of IVIM-DWI and conventional DWI in differentiating ICC and HCC in cirrhotic livers, and to subsequently compare the diagnostic performance of various parameters derived from IVIM-DWI and conventional DWI.

## RESULTS

### Patient characteristics

The final study population for the ICC group were 65 patients (44 men and 21 women; mean age, 54.95 ± 11.83 years; range, 35–80 years) with 68 tumors, and the tumors’ diameter were ranged from 8.7–175 mm (63.44 ± 4.89 mm); the HCC group were consisted of 65 patients (50 men and 15 women; mean age, 52.69 ± 10.86 years; range, 31–78 years) with 68 tumors, and the tumors’ diameter were ranged from 6.5–168 mm (59.60 ± 4.71 mm). For each group, there were 61 patients with Child-Pugh A, 4 patients with Child-Pugh B and no Child-Pugh C patient. No statistical significance was obtained from the patients’ gender (*P* = 0.24), age (*P* = 0.26), nodule size (*P* = 0.57) and Child-Pugh classification (*P* > 0.999) in the comparison of HCC and ICC group.

### Comparison of IVIM-DWI and conventional DWI parameters

**Figure 1:**
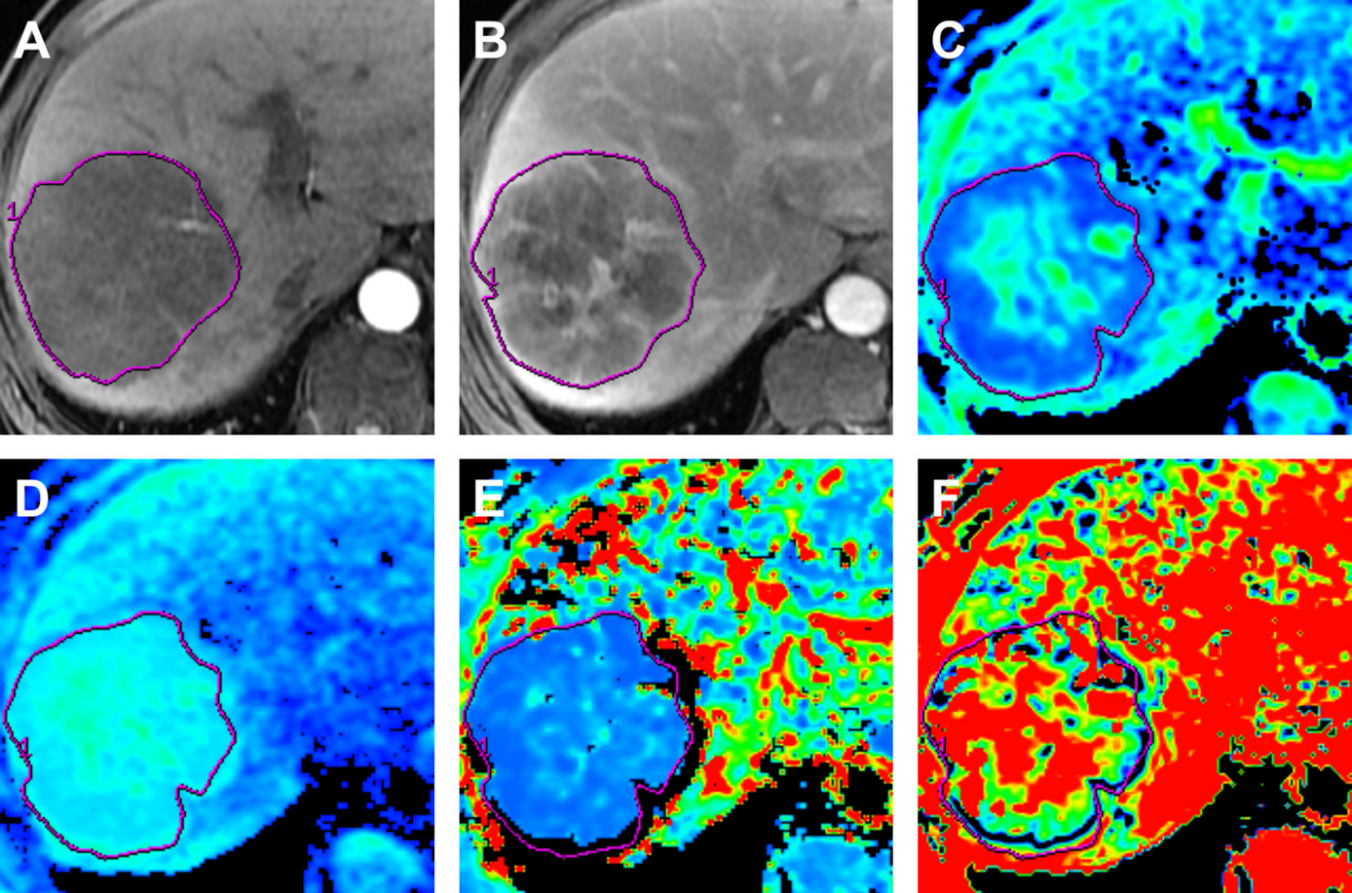
MR images in a 63-year old man with surgically proved ICC (**A**) Arterial phase MR image. (**B**) Portal venous phase MR image. (**C**) Standard ADC map. (**D**) ADC_slow_ map. (**E**) ADC_fast_ map. (**F**) *f* map. The tumor shows peripheral rim-like enhancement on arterial phase MR image and with progressive central enhancement on portal venous phase. The ADC map demonstrates a higher ADC value (1.41 × 10^−3^ mm^2^/s) compared with cutoff value (1.18 × 10^−3^ mm^2^/s), the ADC_slow_ also demonstrates a higher ADC value (1.26 × 10^−3^ mm^2^/s) compared with cutoff value (1.06 × 10^−3^ mm^2^/s).

**Figure 2:**
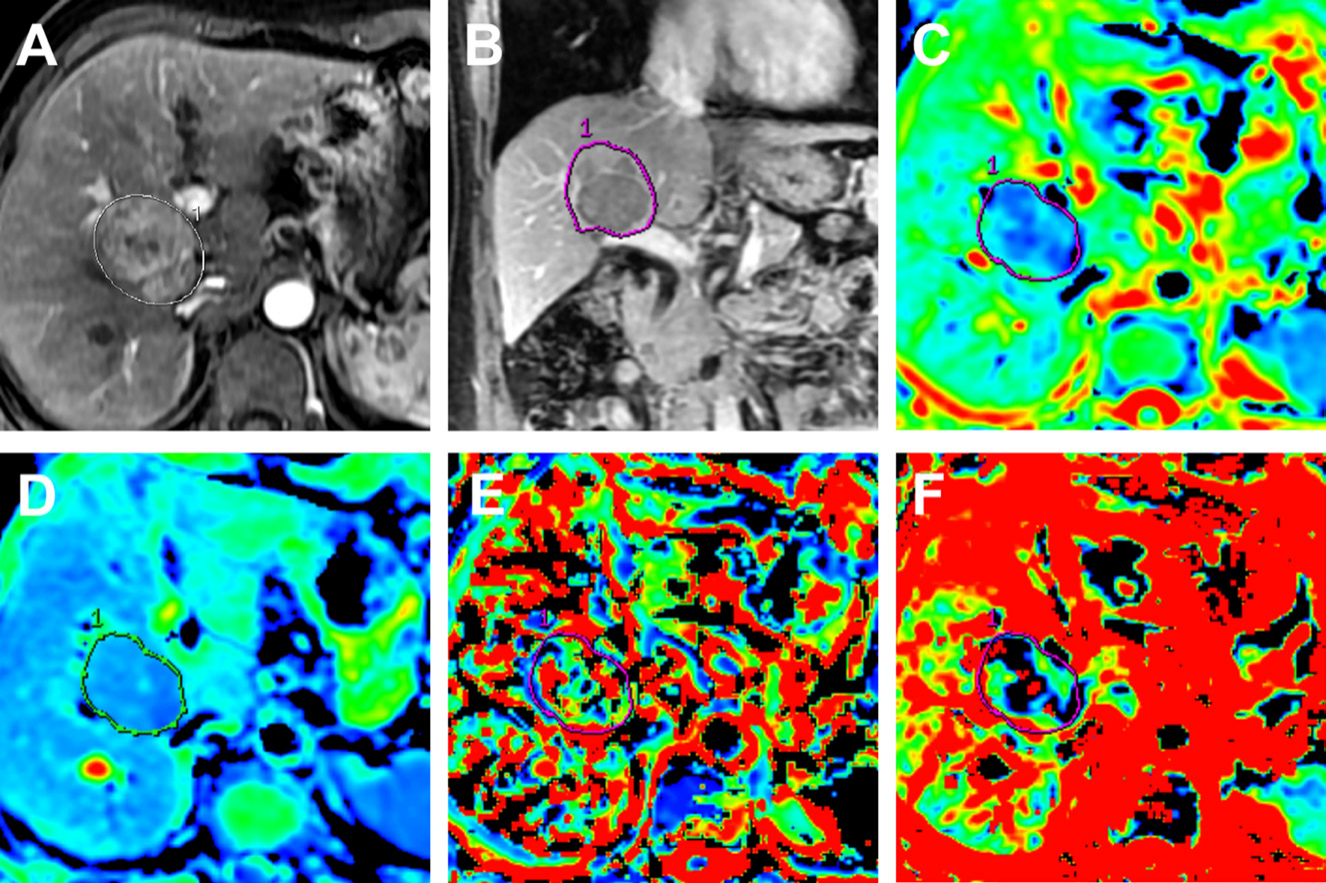
MR images in a 58-year old man with surgically confirmed HCC (**A**) Arterial phase MR image. (**B**) Portal venous phase MR image. (**C**) Standard ADC map. (**D**) ADC_slow_ map. (**E**) ADC_fast_ map. (**F**) *f* map. The tumor shows typically hyperintense on arterial phase MR image and hypointense to liver parenchyma on portal venous phase. The ADC map demonstrates a lower ADC value (0.98 × 10^−3^ mm^2^/s) compared with cutoff value (1.18 × 10^−3^ mm^2^/s), the ADC_slow_ also demonstrates a lower ADC value (0.76 × 10^−3^ mm^2^/s) compared with cutoff value (1.06 × 10^−3^ mm^2^/s).

**Figure 3:**
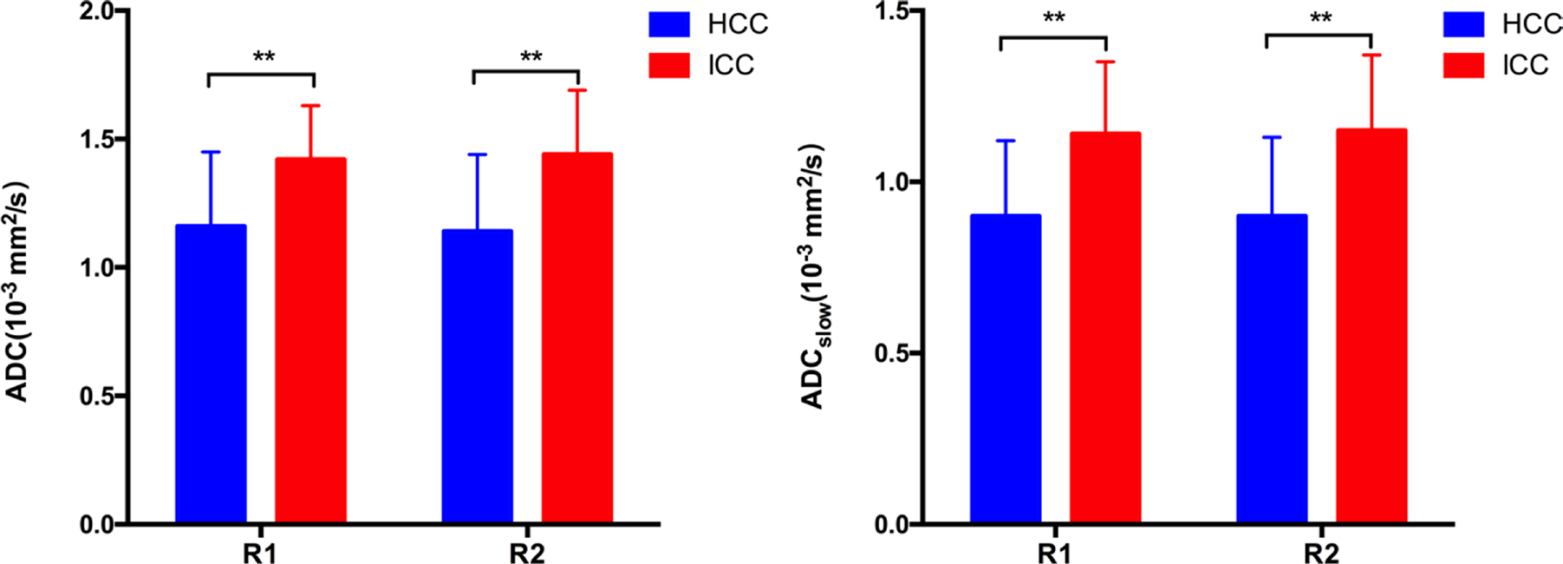
ADC_slow_ and ADC (mean ± standard deviation) value measured by two radiologists for ICC and HCC group R1 = Radiologist 1; R2 = Radiologist 2. The ADC and ADC_slow_ of the ICC group were significant higher than HCC group by both two radiologists. (^**^P < 0.001)

The ADC value (R1, *P <* 0.001; R2, *P* < 0.001), determined by both radiologists, of the ICC (Figure 1) was significant higher than the HCC group (Figure 2). As for the IVIM parameters, the ADC_slow_ (R1, *P <* 0.001; R2, *P* < 0.001) value of the ICC group was significantly higher than HCC, however, the ADC_fast_ (R1, *P* = 0.118; R2, *P* = 0.072) and *f* (R1, *P* = 0.112; R2, *P* = 0.104) value of the ICC group demonstrated a numerical value increasing trend than HCC group but without the statistical significance. The detailed value of the IVIM and DWI parameters of ICC and HCC group were listed in Table 1. Figure 3 shows the quantitative comparison of differences in ADC_slow_ and ADC in the ICC and HCC groups.

**Table 1:** IVIM and DWI parameters of the HCC and ICC group of the two radiologists

Parameters	Observer	HCC	ICC	*t*/*z*	*P*
ADC	R1 R2	1.16 ± 0.29 1.14 ± 0.30	1.42 ± 0.21 1.44 ± 0.25	5.896 6.346	< 0.001 < 0.001
ADC_slow_	R1 R2	0.9 ± 0.22 0.90 ± 0.23	1.14 ± 0.21 1.15 ± 0.22	6.360 6.678	< 0.001 < 0.001
ADC_fast_	R1 R2	16.75 ± 11.53 16.54 ± 11.51	19.92 ± 11.99 20.14 ± 11.66	1.572 1.814	0.118 0.072
*f*	R1 R2	0.37 ± 0.16 0.36 ± 0.16	0.34 ± 0.12 0.33 ± 0.13	1.601 1.637	0.112 0.104

Note: R1: radiologist 1, R2: radiologist 2. HCC: hepatocellular carcinoma, ICC: intrahepatic cholangiocarcinoma, ADC: apparent diffusion coefficient, ADC_slow_: slow ADC, ADC_fast_: fast ADC. ADC, ADC_slow,_ ADC_fast_ are in units of ×10^−3^mm^2^/s, *f* is in units of 100%.

### Diagnostic performance of IVIM-DWI and conventional DWI parameters

**Figure 4:**
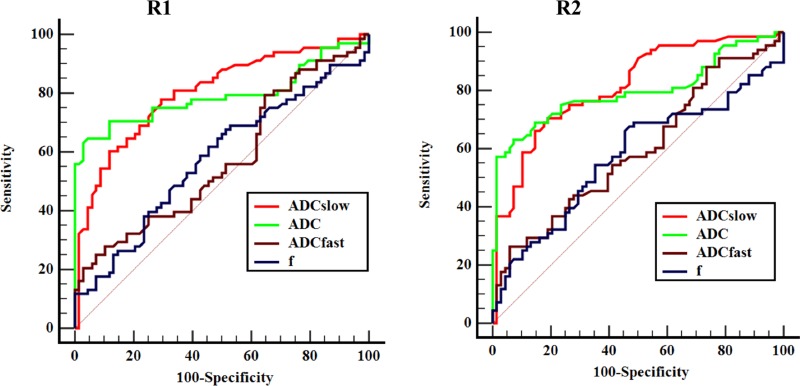
Receiver operating characteristic (ROC) curves of IVIM-DWI and conventional DWI parameters measured by two radiologists for differentiating ICC and HCC R1 = Radiologist 1; R2 = Radiologist 2. Area under curve (AUC) for ADC and ADC_slow_ were higher than the ADC_fast_ and *f* obtained by two radiologists, and the differences were statistical significance.

The receiver operating characteristics (ROC) curves in distinguishing ICC from the HCC group derived from two radiologists of IVIM-DWI and conventional DWI parameters were listed in Figure 4. The ADC_slow_ demonstrated the highest area under curve (AUC) with a value of 0.803 (95% Confidence Interval [CI]: 0.726-0.866) obtained by radiologist 1, and AUC values were 0.792 (CI: 0.714–0.857) for ADC, 0.573 (CI: 0.485 −0.657) for ADC_fast_, and 0.578 (CI: 0.490–0.662) for *f* value. The AUC values of ADC_slow_ was statistical greater than ADC_fast_ (*Z* = 4.003, *P* < 0.001) and *f* (*Z* = 3.769, *P* = 0.002), and AUC values of ADC was also statistical greater than ADC_fast_ (*Z* = 3.986, *P* = 0.001) and *f* (*Z* = 3.048, *P* = 0.002). However, no statistical significant difference was obtained from the AUC values between the comparison of ADC and ADC_slow_ (*Z* = 0.309, *P* = 0.758), ADC_fast_ and *f* (*Z* = 0.092, *P* = 0.927). Additionally, AUC values obtained by radiologist 2 were 0.814 (CI: 0.738-0.875) for ADC_slow_, 0.797 (CI: 0.719-0.861) for ADC, 0.594 (CI: 0.507-0.678) for ADC_fast_ and 0.575 (CI: 0.487-0.659) for *f*. The AUC values of ADC_slow_ was statistically greater than ADC_fast_ (*Z* = 4.071, *P* < 0.001) and *f* (*Z* = 4.087, *P* < 0.001), AUC values of ADC was also statistically greater than ADC_fast_ (*Z* = 3.965, *P* = 0.0001) and *f* (*Z* = 3.232, *P* = 0.001). However, no statistical significant difference was obtained from the AUC values between the ADC_slow_ and ADC (*Z* = 0.517, *P* = 0.605), ADC_fast_ and *f* (*Z* = 0.370, *P* = 0.717). Table 2 shows the sensitivity and specificity of IVIM and DWI parameters at optimal cutoff values in differentiating ICC and HCC groups.

**Table 2:** Sensitivity and specificity of IVIM-DWI and conventional DWI parameters at optimal cutoff value in differentiating the HCC and ICC measured by two radiologists

Group	Observer	Optimal cutoff value	Sensitivity (100%)	Specificity (100%)	Youden index
ADC	R1 R2	1.18 1.14	64.71 (44/68) 57.35 (39/68)	95.59 (65/68) 98.53 (67/68)	0.603 0.559
ADC_slow_	R1 R2	1.06 0.995	77.94 (53/68) 70.59 (48/68)	70.59 (48/68) 80.88 (55/68)	0.485 0.515
ADC_fast_	R1 R2	7.99 8.47	25.00 (17/68) 26.47 (18/68)	92.65 (63/68) 94.12 (64/68)	0.177 0.206
*f*	R1 R2	0.30 0.30	67.65 (46/68) 67.65 (46/68)	48.53 (33/68) 52.94 (36/68)	0.161 0.206

Note: R1: radiologist 1, R2: radiologist 2. ADC: apparent diffusion coefficient, ICC: inter-class correlation coefficient, ADC_slow_: slow ADC, ADC_fast_: fast ADC. ADC, ADC_slow,_ ADC_fast_ are in units of ×10^−3^ mm^2^/min, *f* is in units of 100%.

### Inter-observer reproducibility

**Figure 5:**
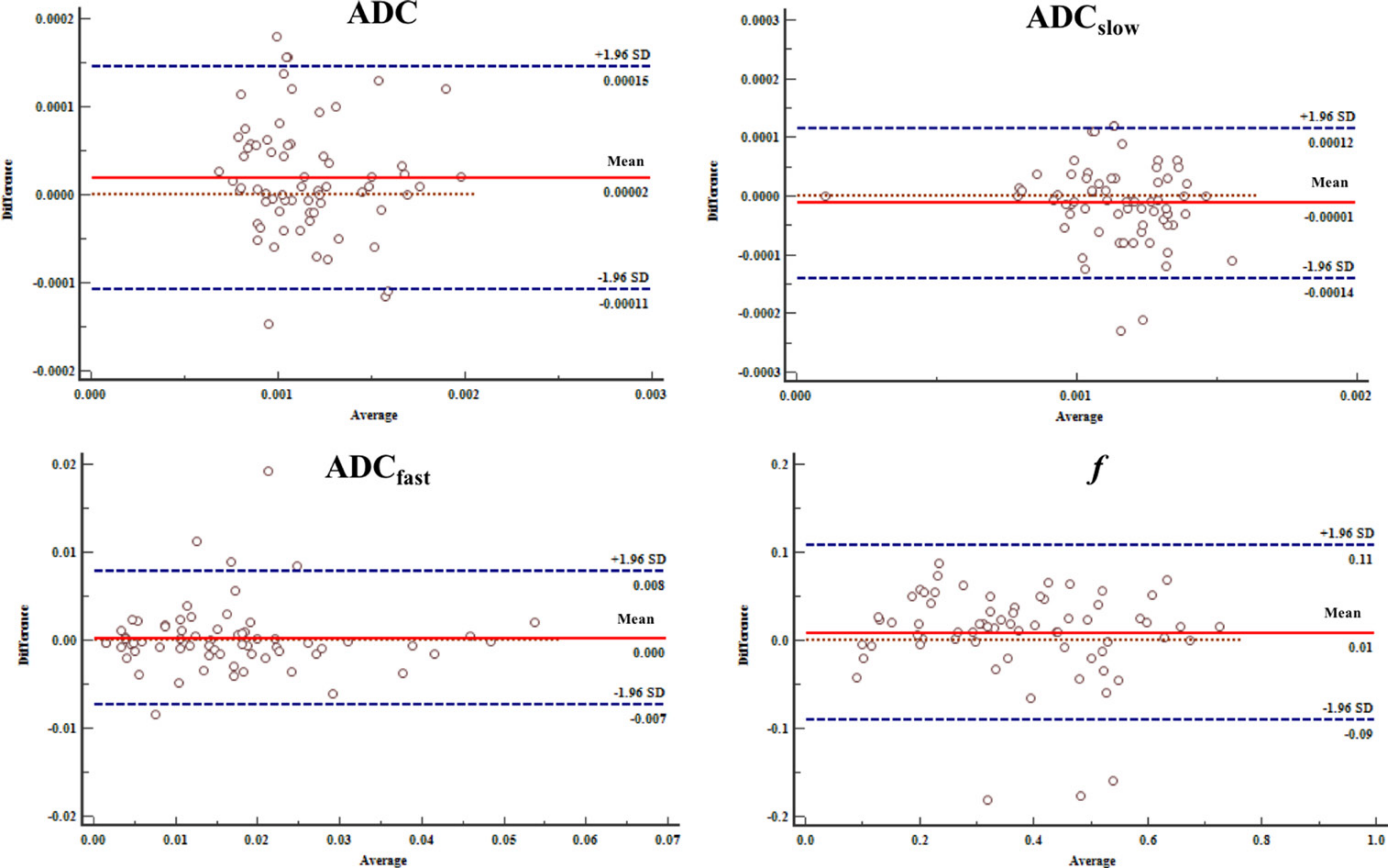
Bland-Altman analysis of differences between two radiologists of the IVIM-DWI and conventional DWI parameters with HCC The differences were relatively small measured by two radiologists toward the parameters.

**Figure 6:**
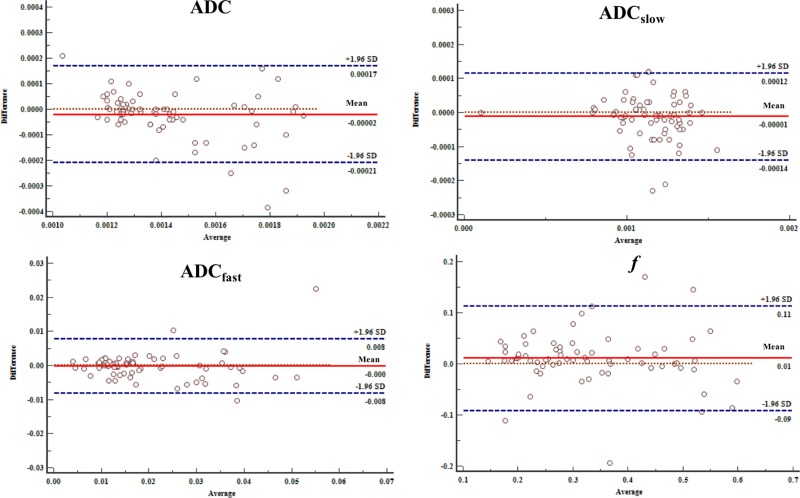
Bland-Altman analysis of differences between two radiologists of the IVIM-DWI and conventional DWI parameters with ICC The differences were relatively small measured by two radiologists toward the parameters.

When looking at the intra-class correlation coefficient between the two observers, the intra-class correlation coefficient values of the HCC group were 0.976 for ADC, 0.960 for ADC_slow_, 0.944 for ADC_fast_ and 0.950 for *f*. For the ICC group, intra-class correlation coefficient values were 0.911 for ADC, 0.952 for ADC_slow_, 0.939 for ADC_fast_ and 0.912 for *f*. The Bland-Altman analysis for inter-observer measurement in the IVIM-DWI and conventional DWI parameters are shown in Figure 5 and 6.

## DISCUSSION

Accurate preoperative differentiation of ICC and HCC has always been limited because of the similar imaging signs depicted by using the usual imaging modalities can not be used to accurately differentiate these entities [14, 15]. HCC usually coexists with liver cirrhosis, whereas ICC is traditionally considered to mainly in the absence of cirrhosis and which used to be regarded as the differential diagnosis point [22, 23]. However, ICC can also be presented with cirrhosis especially with the risk factor of HBV infection, so the differentiation of ICC and HCC can be much more difficult in the cirrhotic livers. Regarding the ICC MR imaging, the ADC value has been used to assess the differentiated grade and tumor response after local-regional therapy [24, 25], and the contrasted enhanced MR has been used to evaluate the enhanced characteristics [26, 27]. However, few studies have determined the feasibility of IVIM in differentiating the ICC and HCC in cirrhotic livers.

In the present study, both ADC and ADC_slow_ showed statistical significance in differentiating ICC and HCC, and the numeric values of ICC was statistical higher than HCC. Histologically, the tumor's cellularity of the HCC was higher than ICC, and high cellularity could decrease extracellular space and limit water diffusion, finally, the value of diffusion coefficient for ADC and ADC_slow_ were decreased. Furthermore, the pathological structures of ICC were mainly consisted of malignant tumor cell, fibrotic tissue and necrotic tissue, the periphery of the tumor were tumor cells and the center of the tumor were mainly fibrotic tissue [28]. The cellularity of ICC is relatively small and the existed tumor cell could arrange into acinar like structures, thus, which could further prompt the water molecular diffusion and increase the ADC and ADC_slow_ value. Previous studies compared the ADC value of ICC and normal hepatic parenchyma and showed that the value of ICC was lower than the normal hepatic tissue [29], and some other studies also reported that the ADC value of the HCC was also lower than that of the relatively low cellularity benign nodules [30, 31]. These current results were consistent with the findings that low cellularity tumor demonstrated a high ADC value compared with the high cellularity tumor.

Our results indicated that the numerical values of ADC were greater than ADC_slow_ in both HCC and ICC groups, and which could be explained by the contribution of perfusion to the diffusion coefficient being removed. In addition, the IVIM-derived ADC_slow_ showed higher AUC value compared with ADC in differentiating ICC and HCC group, and the better diagnostic performance of ADC_slow_ can be explained by the fact that ADC is a non-specific parameter and which could not only influence by the microcirculation related perfusion but also affected by the cellularity related diffusion. Woo et al [32] reported that a high AUC value for ADC_slow_ than for ADC in differentiating high-grade HCC from low-grade HCC. Klauss M et al. [33] also demonstrated a higher AUC value of ADC_slow_ in differentiating HCC and hepatic benign nodules. Thus, ADC_slow_ might be better to categorize the ICC and HCC entities.

It is noteworthy that despite a better diagnostic performance of ADC_slow_ and ADC in differentiating the ICC and HCC group, an overlap of the values still existed in the two groups. This may be related to the following factors. First, the pathological heterogeneity of the tumor. As the recent evidence [28] suggested that the ICC has multiple cellular origins including differentiated hepatocytes, intrahepatic biliary epithelial cells and pluripotent stem cells, the multiple cellular origins may have similar biological behaviors. Thus, from the cell origin and biology perspective, determination of the two kinds of tumor may also overlap. Second, the evaluation of the abdominal MR imaging is hindered by the respiratory motion artifacts and which may also impact on the results. Furthermore, the study cohort was all HBV-related patients with cirrhotic livers, the common pathogenesis of the two groups may also share the common pathological process, so which may also cause the overlap.

Our data demonstrated that the perfusion parameters, ADC_fast_ and *f* were not statistical significantly in differentiating the ICC and HCC group. In theory, the ADC_fast_ is correlated with the average blood flow rate and *f* is associated with the fraction volume of capillary blood flow, which could be used to reflect the vascularity in tissue, and might be helpful in differentiating the ICC and HCC group. Paradoxically, ADC_fast_ and *f* values of the ICC and HCC group showed no statistical difference in our study. The current result may be attributed to the fact that measurement of the ICC and HCC groups were in the setting of liver cirrhosis and which may share the similar blood supply. Previous studies [34, 35] dealing with the enhanced MR imaging showed similar enhancement pattern of ICC compared with HCC, thus, the perfusion related parameters was not helpful in differentiating the HCC-mimicking blood supply of ICC. Yoon JH et al. [30] also reported that ADC_fast_ and *f* can not be used to characterize focal hepatic lesions, Sun et al. [36] reported that the ADC_fast_ and *f* were not useful for assessing the tumor differentiation grade in rectal cancer because of the lower diagnostic efficiency, poor reproducibility and high uncertainty of ADC_fast_ and *f*, which were consistent with our results.

We acknowledge some limitations of our study. First, because of its retrospective nature, there may have been potential for bias in patient selection. Second, the ROIs were mainly selected on the solid parts instead of the whole part of carcinoma, as the heterogeneity of tumor exists, which may lead to selection bias. Thirdly, we did not compare the usefulness of IVIM in the differentiating of ICC and HCC with that of other imaging modalities, such as the dynamic contrast-enhanced MR and enhanced CT, because part of participants did not conduct with enhanced MR or CT examination. Fourth, this is a single-center study with a single MR unit which leaves the question open on the reproducibility. Finally, the ICC and HCC specimens underwent IVIM examination were not included in our study, as which could entirely eliminate the effect of perfusion.

In conclusion, the results of the preliminary study have demonstrated that ADC_slow_ values calculated with IVIM modeling of diffusion weighted imaging and ADC values derived from mono-exponential model demonstrated a superior diagnostic performance than ADC_fast_ and *f*.

## MATERIALS AND METHODS

### Participants

This study was approved by the institutional review board (West China Hospital, Sichuan University), and written informed consent was waived for this retrospective review. This study was conducted in accordance with the Declaration of Helsinki. For the study population, 133 registered patients with ICC on cirrhotic livers confirmed by pathologically were potentially included between January 2014 and March 2017. Among them, 68 subjects were excluded for the following reasons: 1, IVIM examination was not performed (*n* = 12); 2, pathologically combined with HCC and ICC (*n* = 36); 3, the image quality was unsatisfactory (*n* = 7); 4, patients without HBV infection (*n* = 13). For the control group, patients with pathologically proved HCC with HBV-related cirrhosis were one-to-one matched with ICC patients. The matching criteria were: 1, age (± 5 years); 2, HBV-related cirrhosis; 3, number of tumors (single or multiple); 4, tumor size (less than 20 mm, 21–40 mm or ≥ 41mm); 5, Child-Pugh classifications.

### Imaging technique

For all examinations, studies were carried out by using a 3.0 T MR system (Discovery MR750, GE Healthcare, Milwaukee, USA). An eight-channel phased-array torsor coil (GE Medical System) was used for all measurements. The routine MR imaging was performed with a fast spin echo (FSE) sequence with respiratory gating. Fat-saturation axial T2 images were obtained with repetition time/echo time (TR/TE) of 3529/77.6 ms (effective), and the slice thickness was 5.0 mm with gap of 1 mm; field of view, 38 × 38 cm^2^; matrix size, 320 × 320; NEX, 2.0. Axial enhanced T1-weighted images were obtained with TR/TE of 3.9/1.8 ms (effective), and the slice thickness was 5.0 mm with gap of 1 mm; field of view, 38 ×30.4 cm^2^; matrix size, 320 × 160; NEX, 1. The total scanning time for routine sequences were about 15 minutes. The IVIM was performed by using an echo-planner imaging in the axial plane with respiratory gating. The parallel imaging was used and the parameters were: TR/TE, 3750/61.4; field of view, 38 × 28 cm^2^; matrix size, 128×128 and the slice thickness was 5.0 mm with gap of 1 mm. Thirteen b values were from 0 to 1200 sec/mm^2^ (0, 10, 20, 40, 80, 100, 150, 200, 400, 600, 800, 1000, 1200) were used, and the NEX for each b was 2, 2, 2, 2, 2, 2, 2, 2, 4, 6, 8, 10. The total scanning time for IVIM was about 10 minutes.

### Imaging analysis

All the MR images were obtained and transferred to the workstation (Advantage workstation 4.6; GE Medical System). Two independent radiologists (B.S., Y.W., with 25 and 5 years of experience in reading MR images,

$$\frac{\text{S}\left(b\right)}{\text{S}\left(0\right)}=\exp\left(-\text{b}\times \text{ADC}\right)$$

respectively) who were blinded to the histopathological results evaluated the IVIM data. The MR images were anonymized and randomized distributed by a third radiologists (M.W.). The ADC value was calculated by using a mono-exponential model with the equation:

Where S(*b*) represents the signal intensity in the

$$\frac{\text{S}\left(b\right)}{\text{S}\left(0\right)}=f\text{exp}\left(-b\times {\text{ADC}}_{\text{fast}}\right)+(1-f)\text{exp}\left(-b\times {\text{ADC}}_{\text{slow}}\right)$$

presence of diffusion sensitization, and S(0) represents the signal intensity in the absence of diffusion sensitization. For the true ADC (ADC_slow_), pseudo-ADC (ADC_fast_) and perfusion fraction (*f*), the bi-exponential IVIM model was used with the equation:

For every patient, the two independent radiologists placed three region of interests (ROIs) on the solid part of tumor per slice, and three continuous slices according to the largest diameter of tumor were selected for measurement. Identification of selection of the representative tissue for ROIs placement were performed on the DW images (*b* = 0). T2-weighted and dynamic contrast-enhanced T1-weighted images were also used to avoid the hemorrhagic, calcified and necrotic areas. The mean value of ROIs on each parameter map was calculated. The shape, size and positon of ROIs were consistent on each parameter map.

### Statistical analysis

All statistical analyses were performed by using a statistical software package (SPSS19.0 (SPSS Inc, Chicago, IL, USA)). Numerical variance is indicated as the mean and standard deviation. Baseline patient characteristics between the ICC and HCC group were compared with the independent-sample *t* test for the continuous variables and *χ*^2^ *test* for the categorical variables. The mean value of each parameter for each subject measured by the two radiologists were used for statistical analyses, respectively. MR parameters between ICC and HCC were compared with independent-sample *t* test. Receiver operating characteristics (ROC) curves analyses were performed to evaluate the diagnostic performance of each IVIM parameters and DWI in distinguishing ICC from HCC groups, and to determine the optimal parameter for the differential diagnosis. The cutoff point was determined by using the maximized value of Youden index, sensitivity, specificity at the threshold value were calculated. *Z*-test was used to compare the area under ROC curves (AUC) in IVIM and DWI parameters. Intra-class correlation coefficient (ICC) was used to determine the reliability between the two independent radiologists in each parameter, ICC values less than 0.5 are indicative of poor reliability, 0.5–0.75 indicate moderate reliability, 0.75–0.9 indicate good reliability, values greater than 0.90 indicate excellent reliability [37]. Bland-Altman plot was used to evaluate the agreement between the inter-observer measurements. A *P* value of less than 0.05 was considered to indicate statistical significance.
